# Web-based smoking cessation intervention that transitions from inpatient to outpatient: study protocol for a randomized controlled trial

**DOI:** 10.1186/1745-6215-13-123

**Published:** 2012-08-01

**Authors:** Kathleen F Harrington, Julie A McDougal, Maria Pisu, Bin Zhang, Rajani S Sadasivam, Thomas K Houston, William C Bailey

**Affiliations:** 1 Department of Medicine, Division of Pulmonary, Allergy & Critical Care Medicine, The University of Alabama at Birmingham (UAB), 619 19th Street South – OHB 143, Birmingham, Alabama, 35249-7337, USA; 2 UAB Department of Medicine, Division of Pulmonary, Allergy and Critical Care Medicine, 619 19th Street South – OHB 130-B, Birmingham, Alabama, 35249-7337, USA; 3 UAB Division of Preventive Medicine and Center for Outcomes and Effectiveness Research and Education (COERE), 1700 11th Avenue South – MT 628, Birmingham, AL, 35249-4410, USA; 4 Division of Biostatistics and Epidemiology, Cincinnati Children's Hospital Medical Center, MLC 5401, 3333 Burnet Avenue, Cincinnati, OH, 45229, USA; 5 Division of Health Informatics and Implementation Science, University of Massachusetts Medical School – AC7-116, 55 Lake Avenue, North - Room S7-321, Worcester, Massachusetts, 01655, USA; 6 UAB Department of Medicine, Division of Pulmonary, Allergy & Critical Care Medicine, 619 19th Street South – NHB 102, Birmingham, Alabama, 35249-7337, USA

**Keywords:** Tobacco, Smoking cessation, Hospitalized smokers, Study protocol, Internet intervention

## Abstract

**Background:**

E-health tools are a new mechanism to expand patient care, allowing supplemental resources to usual care, including enhanced patient-provider communication. These applications to smoking cessation have yet to be tested in a hospitalized patient sample. This project aims to evaluate the effectiveness and cost-effectiveness of a tailored web-based and e-message smoking cessation program for current smokers that, upon hospital discharge, transitions the patient to continue a quit attempt when home (Decide2Quit).

**Design:**

A randomized two-arm follow-up design will test the effectiveness of an evidence- and theoretically-based smoking cessation program designed for post-hospitalization.

**Methods:**

A total of 1,488 patients aged 19 or older, who smoked cigarettes in the previous 30 days, are being recruited from 27 patient care areas of a large urban university hospital. Study-eligible hospitalized patients receiving usual tobacco cessation usual care are offered study referral. Trained hospital staff assist the 744 patients who are being randomized to the intervention arm with registration and orientation to the intervention website. This e-mail and web-based program offers tailored messages as well as education, self-assessment and planning aids, and social support to promote tobacco use cessation. Condition-blind study staff assess participants for tobacco use history and behaviors, tobacco use cost-related information, co-morbidities and psychosocial factors at 0, 3, 6, and 12 months. The primary outcome is self-reported 30-day tobacco abstinence at 6 months follow-up. Secondary outcomes include 7-day point prevalence quit rates at 3-, 6-, and 12-month follow-up, 30-day point prevalence quit rates at 3 and 12 months, biologically confirmed tobacco abstinence at 6-month follow-up, and multiple point-prevalence quit rates based on self-reported tobacco abstinence rates at each follow-up time period. Healthcare utilization and quality of life are assessed at baseline, and 6- and 12-month follow-up to measure program cost-effectiveness from the hospital, healthcare payer, patient, and societal perspectives.

**Discussion:**

Given the impact of tobacco use on medical resources, establishing feasible, cost-effective methods for reducing tobacco use is imperative. Given the minimal hospital staff burden and the automated transition to a post-hospitalization tailored intervention, this program could be an easily disseminated approach.

**Trial registration:**

Current Intervention Trial NCT01277250

## Background

The health effects of smoking tobacco and their economic impact are well documented [1]. Alabama’s tobacco smoking prevalence rate of 22.5% [2] exceeds the national average of 19.8% [3], with expected higher rates among hospitalized patients [4,5]. Tobacco cessation studies have demonstrated that proactive recruitment of patients for smoking cessation counseling engages a larger percentage of patients, and intensive counseling with at least 1-month follow-up post-hospitalization is effective [6], with 3-months follow-up found even more effective [7]. However, even longer duration counseling in-hospital, without follow-up, was not found more effective [6]. Together, this suggests that, while hospitalization is an opportunity to introduce smoking cessation, post-discharge follow-up is essential.

In *Crossing the Quality Chasm*, the Institute of Medicine began to emphasize that care should not occur just within face-to-face visits but that ‘access to care should be provided over the internet’ to foster continuous healing relationships [8]. Subsequent reports [9-11] and other groups [12], including the Centers for Medicare and Medicaid Services [13], have continued to support the concept of e-Health tools to increase patient access, motivate patients in their care, and re-engineer patient-centered care. Because of time constraints on providers, and with over 70 million Americans using the internet to access health-related information [14], computer- and internet-based tools are becoming widespread. Even in Alabama, with its low ranking on digital access, 61.7% of households have internet access with additional access through work or community resources [15]. Innovative supplemental e-resources are being created to augment brief tobacco-cessation counseling. These new web-based tools/programs present a unique opportunity to enhance continued smoking cessation for hospitalized patients after discharge.

Secure patient-provider e-messaging systems have been found to improve patient satisfaction and increase practice efficiency while maintaining HIPAA standards [16,17]. The strategy of combining e-coaching and computer-automated tailored information has been shown to be successful [18]. Though these innovative web-based tools/programs present a unique opportunity, reports of web-based interventions for hospitalized smokers are limited [6] and no reviews or meta-analyses that included such programs had been found at the time of writing this manuscript [19,20].

In a meta-analysis, 17% of web- and computer-based smoking cessation out-patient interventions were found effective in doubling quit rates at 6 months among general population participants compared to controls [19]. Also, treatment effects for web-based smoking cessation interventions appear fairly stable over time, as a meta-analysis found cessation rates did not dissipate at subsequent follow-ups [21]. While many of the web-based smoking cessation programs are appropriate for any smoker, they may miss an opportunity to address specific relevance of smoking to the smoker’s recent hospitalization and the unique needs of the smoker in care-transition. For this reason, we have enhanced an existing web-based smoking cessation program to address individualized post-hospitalization smoking-cessation needs through e-messages.

## Methods/Design

### Study design

This study uses a randomized controlled two-arm multiple follow-up design to: (1) test the effectiveness of the Decide2Quit intervention against usual smoking cessation care for hospitalized patients transitioning to outpatient care; and (2) determine the cost-effectiveness of each component of a smoking cessation intervention for hospitalized smokers (the Decide2Quit post-hospital intervention and usual care). The primary outcome for effectiveness is self-reported 30-day point prevalence at 6 month post-hospitalization. Secondary outcomes are: biologically confirmed smoking cessation at 6 months; 30-day point prevalence at 3 and 12 months; and 7-day point prevalence at 3, 6, and 12 months. Consecutive abstinence will be assessed using the three 30-day point prevalence data. The cost-effectiveness of the intervention compared to usual care will be determined by considering costs of intervention implementation and costs of healthcare with effectiveness measured in terms of Quality Adjusted Life Years (QALYs).

This study has obtained approval from The University of Alabama at Birmingham (UAB) Institutional Review Board for Human Use (IRB). All study personnel are IRB trained and Health Insurance Portability and Accountability Act (HIPAA) certified.

### Setting and sample

The setting for this study is UAB Hospital, a 1,000 bed state-of-the-art academic center hospital with 43 patient care areas (PCAs). Of these, 27 PCAs serve patients potentially eligible for study participation. The 16 PCAs not included in this study, maternity and palliative care units and some intensive care and psychiatric units (acute, dementia, and adolescent care), are excluded due to the difficulty of obtaining informed consent and/or the unique smoking cessation needs among the patients served. As well, we will over-recruit smokers with pulmonary and cardiac diseases, as this population is of primary interest to the study collaborative. While UAB Hospital has a non-smoking policy, and patients are expected to abstain from tobacco use while hospitalized, non-adherence to this policy has been noted in patients sufficiently mobile to go outdoors.

The UAB Lung Health Center staff receive a daily report of all current smokers admitted to UAB Hospital during the previous 24 h to provide or facilitate the provision of tobacco cessation usual care at bedside (see Table 1 for description of this component). After provision of usual care bedside, all potentially eligible patients admitted to the selected PCAs between July 2011 and May 2013 will be given the opportunity to learn about the study and enroll. To be eligible for the study, a patient must meet the following criteria: (1) over the age of 18; (2) a current smoker defined as at least one puff in the past 30 days; (3) read and speak English; (4) able to provide meaningful responses to the screening questions and to provide informed consent; (5) have an email address and internet access through self or surrogate; and, (6) not have another household member participating in the study. In addition, patients under isolation precautions, except for contact isolation only, are not approached for participation. Criteria 2, 4 and 6 are common to all CHART sites.

**Table 1:** Intervention and usual care contacts by type, timing, and instigator

	**Type of contact**	**Timing**	**Instigator**	
Usual Care - all patients	Admission booklet page on smoking	At admission	Hospital admitting staff	
	Smoking Cessation local resources handout and brief counseling	During hospital stay	Lung Health Center staff	
	Smoking cessation information in discharge packet^a^	At discharge	Hospital assigned nurse	
Additional Components for those randomized to Intervention	Website registration and orientation	Prior to discharge	Hospital Quit Staff	
	Tailored e-mails	Weekly for 12+ weeks^b^	Automated by web-system	
	Telephone call	7 to 14 days post-discharge	Hospital Quit Staff	
	Secure messaging	At will	Participant or Quit Advisor (Tobacco Treatment Specialist)	
	Website use	At will	Participant	

^a^A portion of which is provided optionally at the discretion of the hospital-assigned nurse.

^b^Minimum of 12 weeks, longer if participant changes their readiness to quit status.

### Participant recruitment and randomization to condition

**Figure 1:**
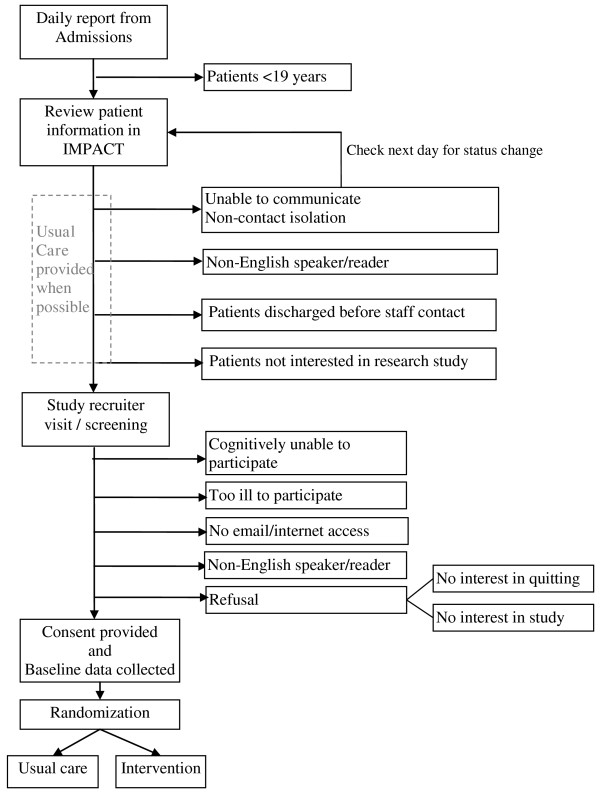
Participant eligibility and recruitment flow.

Study staff verify eligibility with a screening assessment that includes questions about internet access and use of e-mail, the participation of other household members in this study, and mental status (if in question). Eligible patients are provided an overview of the study and a chance to ask questions prior to providing written informed consent. After completion of baseline assessments and listing on the electronic study roll by study staff, patients are randomized to study condition (intervention or usual care). Blocked randomization within each PCA is used to reduce selection and accidental bias and achieve balance in the allocation of participants to treatment arms. The study statistician generated random number lists for each PCA prior to study initiation. The Study Coordinator identifies the participants to randomize to study condition each day based on their order of listing on the study roll and the allocation indicated for the PCA in which they were recruited. As participants are allocated to the intervention arm, the Study Coordinator sends email alerts to designated hospital staff (Quit Staff) that newly randomized patients need to be registered to the intervention website, Decide2Quit. Figure 1 depicts the recruitment and initial study activities flow.

### Intervention description

The intervention includes tailored e-mail messages and an interactive website (Decide2Quit) designed for post-hospitalization smoking cessation which allows secure messaging with a certified Tobacco Treatment Specialist (TTS) (Quit Advisor) and both in- and post-hospital contact by the hospital Quit Staff. The Quit Staff visit patients assigned to the intervention arm at bedside to assist them with registration to Decide2Quit using secure laptops connected to the website via the hospital’s WiFi system. The Quit Staff then orient the participants to the website by displaying general content areas and available quit tools. Participants also receive a booklet illustrating how to access and navigate the website. In addition, this booklet features study contact information for technical problems, blank copies of the website ‘quit plan’ , and a letter for their primary healthcare provider. This letter contains study-related information and requests the healthcare provider’s verbal and pharmaceutical (as appropriate) support of their patient’s quit attempt.

Some patients randomized to the intervention are discharged from the hospital before the Quit Staff can visit them to provide website registration and orientation. The print materials are mailed to these participants and Quit Staff provide telephone assistance with website registration and orientation. The Quit Staff make one follow-up telephone contact, at 7 to 14 days post-discharge, to encourage website use in the context of the primary area of health concern the participant identified at website registration.

### Theoretical framework and intervention e-messages

Two behavioral change theories are the basis for both the website content and e-mail messages: Social Cognitive Theory [22] and the Transtheoretical Model [23]. Two types of email messages are sent: tailored to stage of change and tailored to health concern. Stage of change messages are framed to address where each participant has self-identified along the quit continuum, from no interest in quitting to already quit. For example: those with no interest in quitting receive messages containing information about benefits to quitting and harm reduction; those actively trying to quit receive messages that promote self-efficacy to avoid smoking situations and overcome cravings; and, those already quit receive messages targeting relapse prevention. Participants confirm or change their smoking stage status at each website login. While a set number of messages are available for each stage, when the participant changes their stage, messages ‘start over’ with the messages for the new stage, thereby increasing the total number of messages sent.

The health concern messages, sent weekly for 12 weeks, are based on the primary area of health concern identified by the participant at website registration (cancer, lungs disease, heart disease or stroke, surgery or wound healing, or none of these). E-mail messages provide general information related to the selected health concern, without specifying protected health information. Outcome expectations and other theoretical constructs provide the basis for the messages. For example, someone selecting ‘surgery or wound healing’ would receive the message ‘Quitting smoking allows more blood with oxygen to reach wounds to help them heal faster’ while someone selecting ‘heart disease or stroke’ would receive ‘In just one day of quitting, you will have started to lower your risk of having a heart attack. Every day you stay quit, you lower your risk of having a heart attack more.’ Those choosing ‘none of these’ receive general health messages. Each message ends with promoting website use by providing the link to ‘learn more about ____'. Table 1 summarizes the intervention contacts.

### Web-site content

This intervention website is a modification and refinement of an existing version of Decide2Quit which is the product of two Investigators’ prior NIH-funded research efforts to develop web-delivered smoking cessation systems for patients [24] and providers [25]. The website continues to include an interactive quit-support calculator (questionnaire with feedback focusing on decisional balance, triggers, risks, and smoking-related symptoms), models for communication with health providers and family members, educational materials, a secure messaging system to access a TTS (Quit Advisor), and access to a social network of online smokers (BecomeAnEx). The current site has been expanded and tailored to better serve the unique needs of recently hospitalized adult smokers by providing information on how quitting reduces specific health risks, and the temporary physical and emotional side effects of quitting. The administrative side of the website includes the ability to track patient web activity and smoking status, document follow-up phone calls by the Quit Staff, and monitor participant-generated messages to the Quit Advisor (using the compose function on a secure, HIPAA-compliant messaging system using Secure Socket Layer technology and hosted on a separate server used exclusively for Decide2Quit). Thus, the intervention has both patient-facing and administrative components.

### Identifying and training hospital staff

Meetings with various hospital staff groups led to the identification of respiratory therapists as the most appropriate hospital staff (Quit Staff) for this study given their professional role in lung health and their hospital-wide duties. When the respiratory therapists’ duties preclude assisting with patient registrations, a hospital-paid smoking cessation staff provides this service. Quit Staff provided input into the development of the administrative portal they use to track contact with intervention participants, as well as practical suggestions for how best to implement this intervention in UAB Hospital. Quit Staff training includes a didactic overview of the study, information on specific protocols (registration, orientation, and follow-up telephone contact) and the website, and a hands-on computer training for the website, from both the patient and administrative perspectives.

### Data collection and measurements

Survey data collection takes place at the hospital bedside for baseline and via telephone interview for the 3- , 6- and 12-months follow-up. Additional biologic measures are collected at 6-month follow-up as described below. Tables 2 and 3 describe the measures to be collected at each time point. Measures are noted as Tier 1 (core across all collaborative projects), Tier 2 (optional measures, standard across projects), or Tier 3 (specific to this project). Study staff who conduct assessments are blinded to participant condition and are trained to collect all data uniformly according to written protocols. Data are entered directly into an internet-based survey system using security-enabled laptop computers with either WiFi (at bedside) or hard-wired internet access. All questions require responses by the system to facilitate complete datasets (‘refused’ and ‘does not know’ response options available). Study staff are trained to complete paper copies should there be difficulty in accessing the internet, with the study data manager later entering the data into the database. Some data are acquired from patients’ electronic medical records, study records, or outside sources, as noted in Table 3.

**Table 2:** General assessments and time-points

**Tier**^**a**^	**Measure**	**Details**	**Month**			
			**0**^**b**^	**3**	**6**	**12**
1	Demographics^c^	Age, length of stay, insurance, height, weight, ICD-9 codes, DRGs, Procedure codes, admission type, discharge ‘to’ plan, race, ethnicity, gender, education, marital status, other household smokers,	X			
1	Smoking status	Self-reported - last 7 days		X	X	X
1	Smoking status	Self-reported - last 30 days	X	X	X	X
3	Smoking status	Prolonged abstinence (multiple point prevalence)		X	X	X
1	Quit plan	Post-hospital plans regarding quitting	X			
1	Other tobacco use	Other than cigarettes-last 30 days	X	X	X	X
2	Tobacco dependence	Heavy Smoking Index (2 questions)	X	X	X	X
2	Tobacco cessation treatments	Behavioral and pharmacologic treatments, dose/frequency, length of use	X		X	X
3	Pack-history	Lifetime	X			
3	Longest abstinent period	Lifetime at 0 months; past year at 12 months	X			X
1/3	Satisfaction	Satisfaction with smoking cessation help		X		
3	Quit attempts	Past year (over 24 h abstinent)	X		X	X
3	Social support	Perceived support from significant others to quit/abstain	X		X	X
3	Internet and e-mail use	Frequency and type of use	X			
1	Self-efficacy for abstinence	Single question	X		X	X
3	Self-efficacy for abstinence	SEQ12	X		X	
2	Alcohol use	Audit-C	X		X	X
2	Depressive symptomology	PHQ-2	X		X	X
2	Saliva cotinine, exhaled CO, surrogate corroboration^d^	Validate 7-day smoking status			X	

^a^Tier 1 = common to all CHART trials, Tier 2 = common among some CHART sites, Tier 3 = this site only.

^b^Month 0 = Baseline.

^c^Some data collected after hospital discharge from electronic medical records and discharge summaries.

^d^Randomly selected sample of past-7-days smokers and all past-7-days non-smokers.

**Table 3:** Data collection for cost effectiveness analysis

**Cost**^**a**^	**Tier**	**Data item**	**Description**	**Valuation**			
Cost of usual care	1	LHC staff time	LHC staff time spent at bedside	Average time per patient			
	1	Hospital nurse time	Time spent by assigned nurse assembling discharge packet (containing usual care)	Average time per patient			
	1	Materials	Cost of printing	Actual costs			
Cost of intervention	1	Hospital Quit Staff training	Time of trainers and trainees, materials, and so on	Study records			
	1	Hospital Quit Staff time	Time spent on each patient in preparation, implementation, and documentation of website registration/orientation and 7-14 days post-discharge follow-up call	Average time per patientWeb tracking			
	1	Quit Advisor time	Time spent on each patient in preparation, implementation, and documentation of responses to patient-generated messages	Average time per message			
	1	Patient materials	Brochures, booklets, and so on	Study records			
	1	Web site implementation (not development)	(To be determined, though may contain maintenance, servers, programming, content updating, emailing, and so on)	Study records			
	1	Time of participant on website	Time recorded on website per personal login; number, frequency, and content of messaging to Quit Advisor through the *Decide2Quit* website	Website tracking			
				Month/Source			
				0	3	6	12
Healthcare-related costs	2	Health care utilization	Hospitalizations, outpatient visits, procedures, Emergency Room visits (last 6 months) (No/Yes - # times)	X		X	X
	2	Time cost related to care	Time (hours) off work for participant and others who accompany participant to visits	X		X	X
			Gender-age group average wages for the state	Bureau of Labor Statistics			
	2	Out-of-pocket (OOP) costs	Copayments, deductibles, transportation, parking, meals, and so on (including caregivers, when applicable) (last 6 months)	X		X	X
	2	Costs to health care payers	Reimbursements to health care payers according to type of care	Hospital bills / Medicare reimbursements			
	2	Medicines	Copayments, deductibles, OOP costs (last 6 months)	X		X	X
			Wholesale price for health care payers	Red Book			
Smoking cessation products / programs costs	3	OOP costs	Co-payments, and so on. (last 6 months)	X		X	X
	2	Reimbursements	Reimbursements for health care payers	TBD			
Quality of life	2	EQ-5D-5L [26]		X		X	X
	3	SF-12		X		X	X

^a^All costs will be expressed in the same year of currency.

### Biologic measures

At 6-month follow-up, participants are contacted by telephone and asked about their past-7-days’ smoking status. Participants reporting not smoking in the past 7 days and living within a 40-min commute are asked to come to the clinic for in-person saliva collection, for cotinine testing, as well as a Carbon Monoxide (CO) measure. Those living farther away are mailed saliva collection kits as described in the CHART biochemical verification study (see Riley *et al*. CHART Overview paper). A random sample of self-reported past-7-day tobacco users are asked to provide saliva samples in the same manner as non-smokers. For past-7-days smokers, study staff check a random selection list to determine if the participant is selected to provide a biologic specimen. We anticipate this sample to be about 10% of the past-7-days smokers. At the time of the telephone interview, those participants who are to provide in-person biologic measures are scheduled to come into the clinic within the following 2 weeks. If a participant misses two scheduled clinic appointments, a saliva kit is mailed with follow-up attempts the same as for all mailed kits.

The primary biologic measure is saliva cotinine measurement. Salivary cotinine is considered a very sensitive measure for determining smoking status [27,28]; a level of <15 ng/mL confirms abstinence [29,30]. Exhaled air CO levels in exhaled air are measured with a Bedfont Smokerlyzer; a level of <10 parts per million confirms abstinence [31]. CO is considered the primary biological confirmation measure when the saliva cotinine measure is considered potentially invalid due to current use of nicotine replacement therapy or alternate tobacco products. If a selected participant is unable to attend the 6-month in-person visit, every attempt is made to collect the specimen by mailed saliva sample kit or home visit (or other mutually agreed upon meeting place) by study staff. When no biological measure can be acquired from a participant, study staff attempt corroboration of self-reported smoking status from another person (contact information and permission to contact provided by participant at previous contact).

### Participant incentives

Participants are provided checks as incentives for completion of the follow-up data collection portion of this project to compensate for their time and effort. For survey completion: a $20 check is mailed after the 3-month telephone follow-up and $25 checks are mailed after each of the 6- and 12-month follow-up. For those providing saliva samples: those providing saliva samples in the clinic receive a $100 check at the end of visit, while those providing mailed samples will be sent a $75 check upon receipt of the mailed saliva sample.

### Process measures

**Figure 2:**
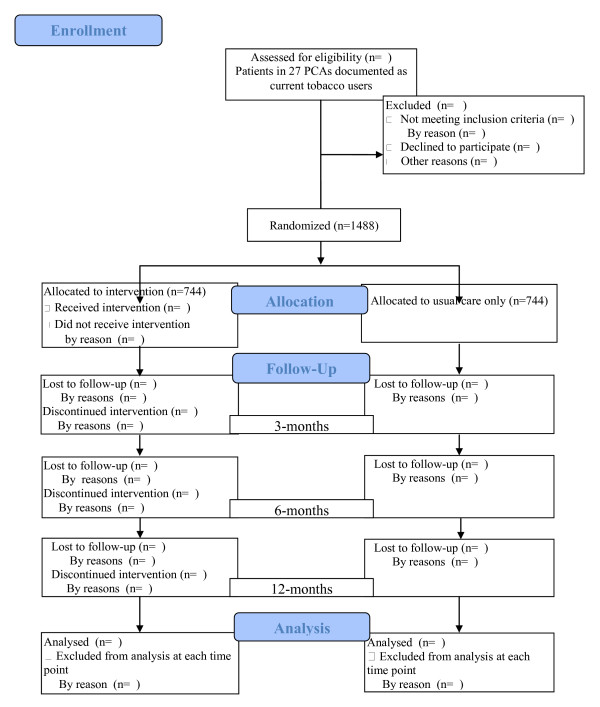
Study flow diagram from enrollment to analyses.

To ensure that the study protocol is implemented with fidelity, systems- and records-based data are collected on an ongoing basis. Process measures fall into three areas: (1) recruitment and retention; (2) intervention delivery and dose; and (3) staff productivity. Study investigators review reports monthly to monitor study activities and make recommendations for remediation as needed. Reports include the proportion of patients participating (or lost) at each level of screening and study activity (Figures 1 and 2). Intervention delivery and dose data collected include website log-ins, tailored e-messages sent, secure messages generated, and post-hospitalization telephone call receipt. Quit Staff manually enter the telephone call information into the website’s administrative portal while all other data are collected automatically by the website system. Staff productivity is monitored to ensure both protocol fidelity and timely completion of the study. These data include contact rates by the Quit Staff (bedside/telephone registrations and follow-up phone calls) and individual recruitment and retention rates for each study staff. Ongoing performance feedback from the Study Coordinator is provided to staff with appropriate recommendations.

### Sample size estimate

We have estimated the total number of participants recruited during the 22-month recruitment phase of this study as 1,488 based on published and UAB-specific information. Given published findings that smoking rates among hospitalized patients exceed general population rates [4,5,32-35], the estimated current rate of smoking in Alabama (22.5%) [2], and the UAB Hospital 2009 data (24% to 32%, depending on the ICD-9 codes used to define current smokers), we estimate that at least 24% of UAB Hospital inpatients will be current smokers. Among hospitalized smokers in other studies, 60% to 82% desired to quit and expressed willingness for post-hospitalization smoking cessation contact [4,33,36]. Almost 62% of Alabama households have internet access [15]; we anticipate 50% of our hospitalized patients who smoke will have internet access, given the likelihood of being from a lower socioeconomic status. Based on current admission trends, we expect an average of 10 new patients to meet eligibility criteria on a typical day within the 27 PCAs targeted, with at least half willing to receive information about the study. It is anticipated that the study population’s demographics and reasons for hospitalization will mimic the general population of hospitalized smokers at UAB Hospital (based on 2009 hospital data): 60% male, 43% African American, 1.5% Hispanic, 1.3% Asian, and <1% of other racial/ethnic background, with approximately 17% having acute myocardial infarction (AMI), pneumonia, and/or congestive heart failure (CHF).

### Power estimate

Power is estimated for the primary outcome: differences in self-reported smoking abstinence rates between the two treatment conditions at 6 months post-hospitalization based on 30-day point prevalence. Rates for smoking cessation reported in the literature for ‘usual’ post-hospitalization care are 5% to 15% at 6 months and 7% to 10% at 1 year [37]. With 1,488 participants, 744 assigned to each treatment condition, using 12% as an estimated rate for the comparison group, this study will be powered (β = 80%-type II error, α = 0.05-type I error) to detect a difference of 5.12% between the two treatment conditions (two-sided). For the intervention condition, a sub-analysis between the group with one of three diagnoses (CHF, AMI, or pneumonia-expected *n* = 256) and the group with all other diagnoses (expected n = 488) will have sufficient power (β = 80%-type II error, α = 0.05-type I error) to detect a difference in tobacco abstinence rates of 9.4% (two-sided), assuming the three-diagnoses comparison group has a 25% quit rate (two-sided). Note that, as the smoking cessation rate increases for the comparison group, a larger difference is necessary between groups to achieve a specific level of power, which is why a higher rate is used for power computation. The reverse of this is true as well: as the comparison group rate decreases, a smaller difference between groups achieves the same level of power.

### Data analyses

#### Primary and secondary outcomes analyses

The study analyses will be conducted using the most up-to-date version of SAS (SAS Institute, Cary, NC, USA) and *P* ≤ 0.05 will be considered significant for all analyses, except where Bonferroni correction for multiple comparisons is necessary. Baseline characteristics of both groups will be compared to ensure that random assignment results in comparable groups. Any factors not found balanced across randomization groups will be adjusted for in subsequent models. Continuous variables will be compared using a two-sample t-test, while a chi-square test of association will be used to compare categorical variables.

The primary outcome for this study will be self-reported 30-day tobacco abstinence rates at 6-months follow-up. For missing outcome data at the 6- and 12- months follow-ups, we will impute missing data using a propensity-based multiple imputation method [38] that has been agreed upon for the collaborative [39]. This approach assumes that non-response is random which is more robust than assuming all non-responders are smokers [40]. Using baseline and any additional available data (for example, 3-month survey), we will use a logistic regression model to estimate the propensity to respond to the follow-up survey for each hospital stratum and treatment arm combination. Within each combination we will stratify further based on values of the estimated propensities and then impute a value for the outcome for each non-respondent by randomly sampling from the respondent values within their sub-stratum. Multiple datasets will be created and the point estimates and the estimated standard error from each dataset will be combined to arrive at a single point estimate, its estimated standard error, and the associated confidence interval or significance test [40]. Given the potential for missing data not-at-random, we will further implement a pattern-mixture analyses [41] based on content experts and the observed patterns of missing data.

We will also run a responders-only analysis for descriptive purposes. A two-sample test for the difference in proportions will be used to test whether the quit rates are different between the two treatment conditions (two-sided) and, if indicated, whether the Decide2Quit intervention is effective in promoting smoking cessation compared to the usual care group (one-sided). We will conduct this same statistical analysis for secondary outcomes.

Several covariates may affect the relationship between the intervention effect and the quit rate. Potential covariates include age, tobacco dependence, major health issue, number of diagnostic codes assigned at hospital discharge, race, smoking history, alcohol use, depressive symptoms, social support, use of other smoking cessation programs, and self-efficacy. Because participants are randomized to treatment arm, these potential covariates will theoretically be balanced across the two arms. However, the strength of the association between each of these covariates and the quit rate will be assessed in a logistic regression model. This analysis will provide the variables that are predictive of smoking cessation among all smokers and can be repeated to test for predictors among the three diagnostic groups (AMI, pneumonia, and CHF) *vs* all other diagnostic groups. Secondary exploratory analyses are planned to investigate a range of broader issues related to smoking cessation. An analysis similar to the primary analysis will test potential differential effects of the Decide2Quit intervention among diagnostic groups by comparing those patients with AMI, CHF, and/or pneumonia to patients not having these diagnoses. A third set of tests of proportions will compare each of the three diagnostic groups of interest in the intervention condition to their matching group in the usual care condition (although power will be limited due to small sample sizes). The relationship between dose and smoking cessation will be examined using logistic regression tests, as we expect dose will not be normally distributed.

#### Cost-effectiveness analyses

The cost-effectiveness will be conducted from the perspectives of the hospital, health care payers, patients, and society. Analyses will be conducted for the short-term of the trial and for the lifetime of participants using modeling techniques and data from the literature, using the established cost-effectiveness analyses measures common to participating CHART projects. Effectiveness will be measured using Quality Adjusted Life Years (QALYs) which will be calculated using the EQ-5D-5L [26]. See Table 2 for all measures related to cost-effectiveness. Incremental cost-effectiveness ratios (ICERs) will be calculated by dividing the net cost of Decide2Quit by its effectiveness, that is, QALYs saved by the intervention. ICERs will be calculated if Decide2Quit is effective in improving QALYs and is more expensive than usual care. Ratios will be compared to others published in the literature to determine if the Decide2Quit intervention is cost-effective. ICERs do not need to be calculated if Decide2Quit is cost-saving, that is, more effective and not more costly than usual care. To examine the robustness of and the impact of parameter uncertainty on these results, univariate and multivariate sensitivity analyses [42] will be used.

In addition, a non-parametric bootstrap method [43-46] will be used to sample with replacement costs and outcomes from usual care and intervention arms. In cost-effectiveness analyses, non-parametric bootstrapping is one of the methods used to allow the comparison of arithmetic means of cost data, which usually are heavily skewed to the right. In the case the cost data are distributed normally, we will use parametric statistical testing. The bootstrapping also is used to examine uncertainty in cost-effectiveness analyses. Relying on a conventional confidence interval of the cost-effectiveness ratio is complicated by the fact that a positive ratio indicates a case of the intervention being more effective and more costly than the comparison (and thus may be cost-effective) and indicates a case of an intervention being less effective and less costly than the comparison. A depiction of cost and effect differences of the bootstrap samples on a cost-effectiveness plane will allow us to better consider these cases and understand the variability around the ratio. The difference in costs and outcomes of each bootstrap sample, obtained by repeating the procedure 1000 times, will be plotted in a cost-effectiveness plane. An acceptability curve then will be obtained by considering the proportion of bootstrap replications for which the CE ratio falls below each possible value of cost per QALY, including the commonly used $100,000/QALY. This information will allow a better understanding of the probability of the Decide2Quit intervention being cost-effective.

## Discussion

There is considerable research on smoking cessation among outpatients and among inpatients with respiratory or cardiac disease. However, for general inpatient smokers, there is little information on programs that transition to post-hospitalization and most have follow-up limited to less than 6 months. Rates of smoking cessation immediately post-hospitalization are high, but relapse rates are also high [47]. Conceptually, relapse prevention should begin in the hospital and continue after discharge, as the crucial time for relapse occurs 1 week after quitting [48]. For many patients, this is concurrent with discharge and the return to a smoking-related routine with deeply engrained smoking-related behaviors [6]. Despite this knowledge, cessation interventions designed to transition patients from hospital to home have not widely diffused into usual clinical practice. This study will add to the limited literature about smoking cessation in the general inpatient population as well as smoking status at 12 months post-hospitalization.

Identifying appropriate hospital staff to register patients for a smoking cessation program was a challenge in the current healthcare system. A strong interest in tobacco cessation and access to and familiarity with all areas of the hospital are considered essential. As hospital resources, environments, and staffing vary, different hospital personnel may be identified as most appropriate. After discussions with personnel on many levels and areas of this hospital, respiratory therapists were identified as the best fit. Regardless, having committed champions among the hospital staff, or even better dedicated hospital staff, is imperative for successful implementation and the institutionalization that leads to program sustainability.

Given the unknown validity of self-reported health behaviors and the expense and difficulty of collecting biological specimens, this study also aims to determine the validity of self-reported smoking status for both self-reported quitters and non-quitters post-hospitalization. Participants are not informed of their selection for biological confirmation until after their quit status is assessed via telephone. We anticipate these findings will provide an algorithm for estimating true rates of smoking status from self-reported rates in future studies, increasing confidence in findings while reducing costs and allowing more precise measures of cost-effectiveness.

A limitation of this study is that the intervention is designed for the general medical or surgical patient. We are not involving adolescents, acute psychiatric patients, and maternity patients as these individuals have unique needs. Ideally, we would include all smokers hospitalized within our medical hospital complex, to be able to meet the needs of every patient in the hospital population; however, given resource and time constraints, it is not feasible for this study. In the future, this website intervention could be expanded, similar to how this study’s website is an expansion of one originally developed for outpatients, to include messages and web pages tailored to meet the needs of these groups. As well, it could be tailored to specialty hospital populations. Another limitation of this study is potential selection bias given that a large proportion of our potential participant pool does not have internet access, a requirement for study participation. Those on the other side of the ‘digital divide’ may vary from our study sample in terms of smoking habits, concomitant tobacco use or other salient attributes. This limits the generalizability of the findings to this study to the proportion of the hospitalized population with internet and e-mail capability.

We have developed a tailored web-based program in an effort to apply these new e-health tools to facilitate the transition of the hospitalized smoker to the outpatient arena. This study will examine the effectiveness and cost-effectiveness of this smoking cessation program that is facilitated by hospital staff and links the discharged patient to a certified TTS and the intervention program for as long as needed. If this program proves to be effective and cost-effective, it will be an approach that could easily be adopted by other hospitals.

## Trial status

This study began recruitment July 17, 2011 and will continue to enroll participants until May 2013.

## Abbreviations

AMI: Acute Myocardial Infarction; Audit-C: Alcohol Use Disorders Identification Test; CHART: Consortium of Hospitals to Advance Research on Tobacco; CHF: Congestive Heart Failure; CO: Carbon Monoxide; HIPAA: Health Insurance Portability and Accountability Act; HSI: Heavy Smoking Index; ICERs: Incremental cost-effectiveness ratios; IRB: Institutional Review Board for Human Use; NIH: National Institutes of Health; OOP: Out of Pocket; PCA: Patient Care Area; PHQ-2: Patient Health Questionnaire-2; QALYs: Quality Adjusted Life Years; SAS: Statistical Analysis Software; SEQ12: Smoking Self-Efficacy Questionnaire; SF-12: Short Form Health Survey (12-item); TTS: Tobacco Treatment Specialist; UAB: University of Alabama at Birmingham.

## Competing interests

The authors have no competing interests, either financial or otherwise.

## Authors’ contributions

TKH and RS designed the website, participated in the study design, and helped draft the intervention description section of this manuscript. MP designed and drafted the cost-effectiveness methods. BZ ran the power analyses and designed the primary analyses methods. JAM participated in the design of some study methods and helped draft the manuscript. KFH and WCB conceived the study, developed the design, and drafted sections of the manuscript. All authors read and approved the final manuscript.

## Authors’ information

Dr Houston received previous NIH-funding (R01CA129091 and R01DA017971) to develop the original Decide2Quit website intervention described herein. His experience with these previous efforts and ongoing internet-based tobacco interventions are influential in the design of this current study’s Decide2Quit intervention website.

## Funding

This work is funded by the National Institute on Drug Abuse at the National Institutes of Health (NIH) through cooperative agreement number 1U01DA031515.
